# Myxoid Liposarcoma: A Case Report of a Rare Location in the Abdominal Wall

**DOI:** 10.7759/cureus.8715

**Published:** 2020-06-20

**Authors:** Marouane Harhar, Abdelbassir Ramdani, Tariq Bouhout, Badr Serji, Tijani El Harroudi

**Affiliations:** 1 Surgical Oncology, Mohammed VI University Hospital, Regional Oncology Center, Oujda, MAR

**Keywords:** myxoid liposarcoma, rectus abdominis muscle, soft tissue tumours

## Abstract

Liposarcomas are considered to be the most common soft tissue sarcomas and have five histological subtypes. The myxoid subtype often occurs in the lower limbs and the retroperitoneum; however, the abdominal wall location is extremely rare. The clinical presentation and radiological findings are non-specific. Wide local excision with a minimum margin of 3 cm remains the mainstay of treatment to prevent local recurrences. We herein report a rare location of myxoid liposarcoma in the abdominal wall.

## Introduction

Liposarcomas (LPSs) represent the most common soft tissue sarcomas [1]. Five histological subtypes of LPS have been described as follows: well-differentiated, myxoid, round cell, dedifferentiated, and pleomorphic variants. This fat tumor, of ubiquitous localization, commonly appears as a slowly enlarging mass with a misleadingly benign appearance. Most myxoid LPSs occur in the lower limbs, such as the thighs, and in the retroperitoneum [2]. The objective of this report is to present an extremely rare case of abdominal wall myxoid LPS.

## Case presentation

**Figure 1:**
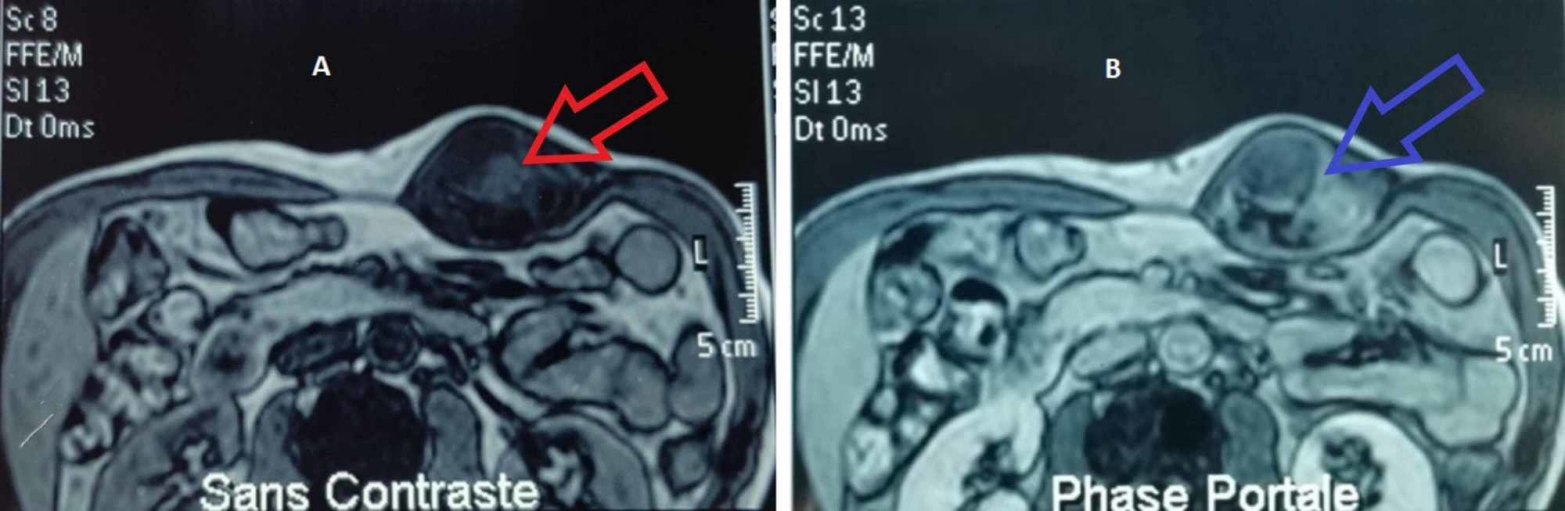
(A) Abdominal MRI showing a hypointense tumor (red arrow) on T1-weighted image. (B) The gadolinium contrast enhancement image shows a predominantly adipose mass containing nodular non-lipomatous components (blue arrow).

A 43-year-old man presented with an abdominal wall mass of nine months duration, increasing rapidly in size without any other associated symptoms. On physical examination, the mass was located in the left abdominal wall. It was soft and mobile, and had a diameter of 9 cm. An abdominal MRI with intravenous contrast revealed a heterogeneous mass in the left rectus abdominis muscle tumor lesion enhanced early and continuously in the late phase, measuring 88 x 65 x 52 mm (Figure 1). A thoracic and abdominopelvic computed tomography (CT) scan was performed, which did not reveal any distant metastasis.

**Figure 2:**
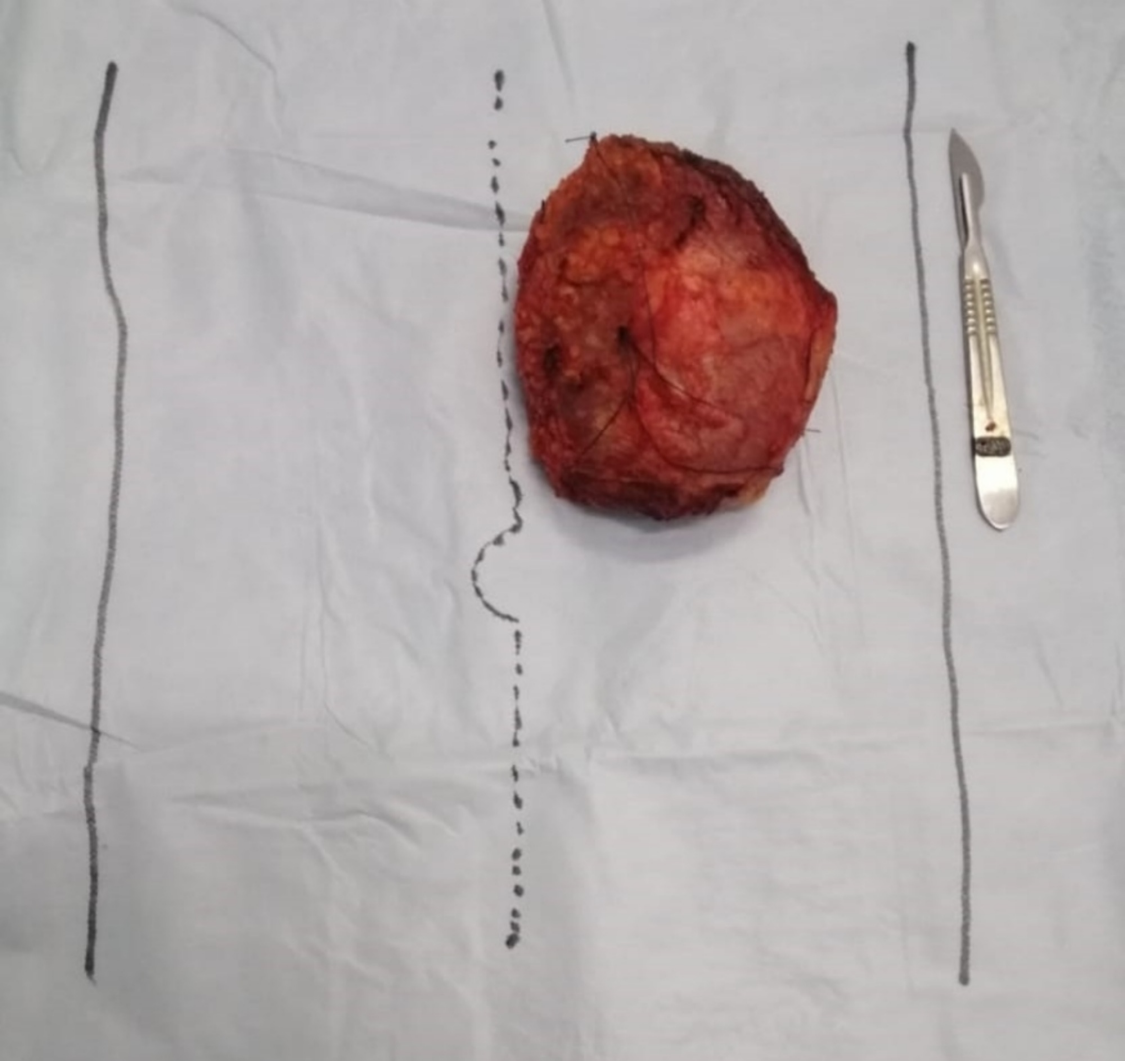
Image of the removed surgical specimen.

Biopsy with Tru-Cut® (Merit Medical Systems Inc., South Jordan, UT, USA) of the mass was consistent with an angioleiomyoma. Immunohistochemistry staining was performed, which revealed the following: CD34 (-), vimentin (-), myogenin (-), and S100 (-). We decided to perform a large excision of the parietal tumor with clear surgical margins followed by the repair of the fascial wound using a nonabsorbable mesh (Figure 2).

The mass was roundish, measuring 9 cm. The gross section showed a yellowish myxoid tumor well-circumscribed capsulated with the following resection margins: 0.2 cm for the deep limit, 0.5 cm for the internal limit, 0.3 cm for the external limit, 0.4 cm for the upper limit, and 0.3 cm for the lower limit.

**Figure 3:**
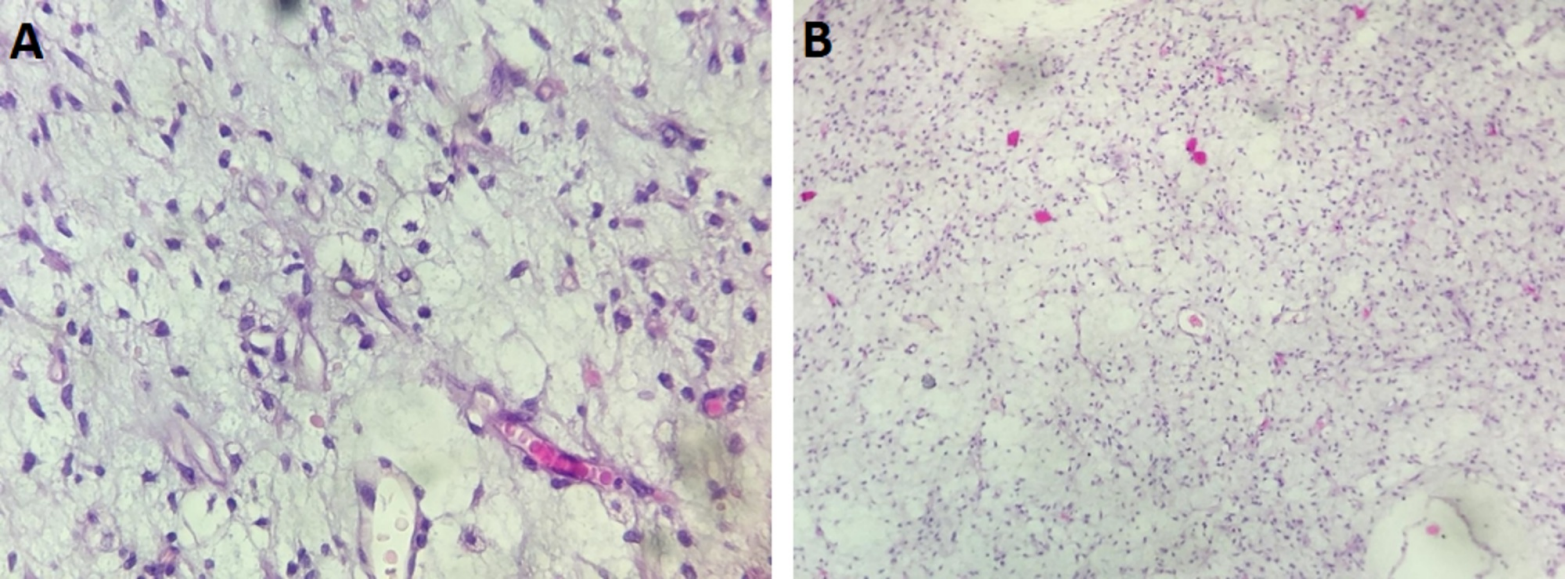
(A) Micrograph showing a proliferation made of lipoblasts deposited on a myxoid stroma. Narrow capillaries are also appreciated in this picture (HE, 400x). (B) The proliferation is hypocellular, and the stroma is myxoid (HE, 100x). HE, hemotoxylin and eosin

The histological examination and immunohistochemistry stainings (PS 100 [+], CD34 [+]) confirmed the diagnosis of myxoid LPS (Figure 3). After a multidisciplinary consultation meeting, the patient was referred to the radiotherapy department for the initiation of adjuvant radiotherapy. The patient was symptom-free, with good wound healing and no recurrence after six months of surgery.

## Discussion

The LPS is one of the most frequent soft tissue sarcomas found in adults. The main site of origin is the thigh (13-60%) and rarely the retroperitoneum [3]. The abdominal wall location is extremely rare and exceptional. The incidence of myxoid LPS is high during the fourth and fifth decades of life, and there is no gender predilection.

The most common histological subtype is myxoid LPS (56.2%) followed by well-differentiated LPS (also known as atypical lipomatous tumor) (21.9%), pleomorphic LPS (17.8%), dedifferentiated LPS (6.8%), and round-cell LPS (4.1%) [4].

Characteristic genetic alteration for myxoid/round-cell LPS is classically a t(12;16)(q13;p11) or t(12;22)(q13;q12) translocation found in more than 95% of myxoid/round-cell LPS, whereas well-differentiated/dedifferentiated LPS is related to the amplification of the 12q13-15 region that comprises the *MDM2* and *CDK4* genes [5].

In our case, the mass was thought to be benign pre-operatively; therefore; an accurate diagnosis was needed. The suspicious abdominal masses should undergo a biopsy prior to surgical resection in all cases. The biopsy usually has a sensitivity of 80 to 95% to define the presence or absence of malignancy [6]. The most suitable method is the core needle biopsy of the tumor using one or more punches. According to a study published by Strauss et al., the tumor subtype was accurately assigned in 89.5% of benign tumors and 88% of sarcomas using core needle biopsy [7]. It is preferred to determine the nature of the tumor using biopsy beforehand in order to help surgeons evaluate the risk and difficulty involved.

The management of myxoid LPS needs to be discussed over multidisciplinary meetings including surgeons, oncologists, and radiotherapists.

The gold standard treatment of abdominal wall sarcoma consists of the en bloc resection of the lesion including 3 cm as a minimum margin of free tissue, involving the entire width of the abdominal wall. If there is tumor adherence or invasion, this resection must also involve adjacent structures or internal organs [8,9]. Abdominal wall reconstruction can be performed using myocutaneous flaps or synthetic mesh, or a combination of both methods [8,10]. In our case, the tumor was giant and the patient underwent en bloc resection; the margins of free tissue were <1 cm. There are some reports that demonstrate that myxoid LPSs are radiosensitive whether the radiotherapy is delivered pre-, intra-, or post-operatively. The optimal radiation dose is 50  Gy in the neoadjuvant setting and 60  Gy in the adjuvant setting [11].

Well-known negative prognostic factors for abdominal wall LPS are age (>45 years), tumor size (>10 cm), high histological grade, and a high percentage of round cells and positive resection margins. On the other side, large and extensive surgical removal has a significant effect on the survival rate [12,13]. Overall, myxoid/round-cell subtypes have a five-year survival rate of 60% to 90% [14].

## Conclusions

Myxoid LPS of the anterior abdominal wall is an extremely rare tumor. The suspicious abdominal masses should undergo a biopsy prior to surgical resection in all cases. Wide local excision of the tumor remains the mainstay of treatment with a minimum margin of 3 cm in light of their tendency to occur locally.
